# Supplementary material for: Proinflammatory polarization of engineered heat-inducible macrophages reprogram the tumor immune microenvironment during cancer immunotherapy
Source: Nat Commun. 2024 Mar 15;15:2270. doi: 10.1038/s41467-024-46210-1 (PMC10943244; doi:10.1038/s41467-024-46210-1)
Supplement: Supplementary file 1 — Supplementary Information [file 41467_2024_46210_MOESM1_ESM.pdf]

Supporting Information for

Proinflammatory Polarization of Engineered Heat-Inducible Macrophages Reprogram the Tumour Immune Microenvironment During Cancer Immunotherapy

*Yanan Xue [1,2,†], Xiaojie Yan [2,3,†], Da Li [1,†], Shurong Dong [4], and Yuan Ping [1,2,3]**

[1] Sir Run Run Shaw Hospital, School of Medicine, Zhejiang University, Hangzhou, 310016, China

[2] College of Pharmaceutical Sciences, Zhejiang University, Hangzhou, 310058, China

[3] Liangzhu Laboratory, Zhejiang University, Hangzhou 311121, China

[4] College of Information Science and Electronic Engineering, Zhejiang University, Hangzhou, 310027, China

† Equally contributing authors.
* Corresponding author. Email: pingy@zju.edu.cn (Y.P.)

Supplementary Figures

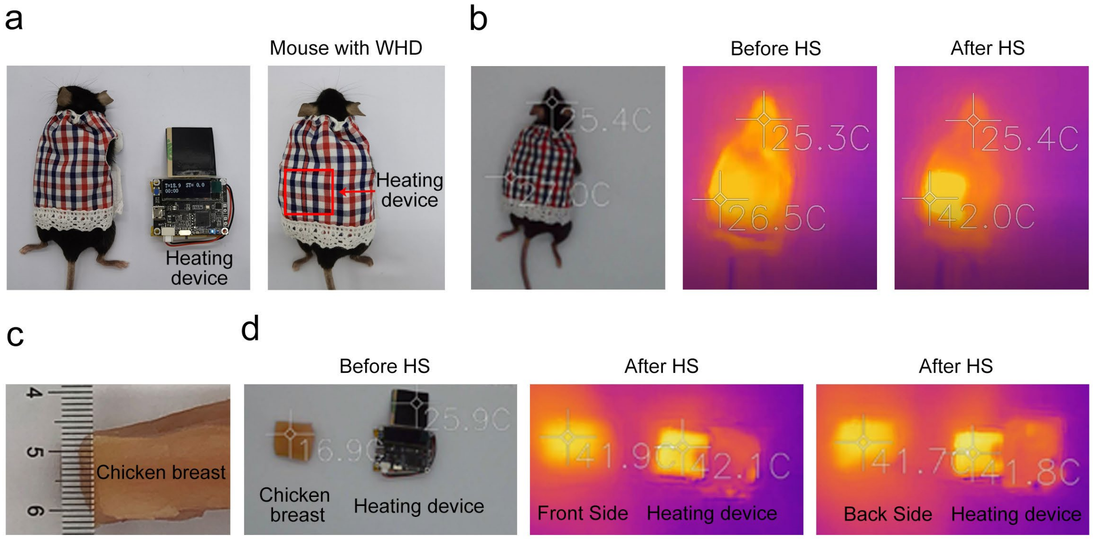

Supplementary Fig. 1 Characterization of the iWarm. (a) The prototype of iWarm. (b) The locoregional temperature of the mouse back after turning on iWarm monitored by thermal imaging equipment. (c) The thickness of the chicken breast. (d) The temperature of contact surface (front side) and the non-contact surface (back side) before and after iWarm-mediated temperature elevation monitored by a thermal imaging camera. This experiment was repeated three times independently with similar results.

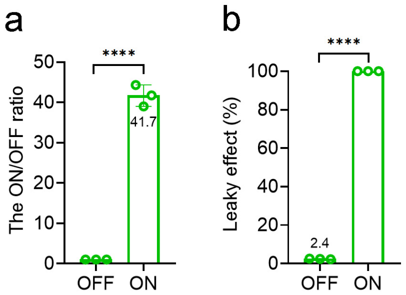

Supplementary Fig. 2 The ON/OFF ratio (a) and the leaky effect (b) of the gene circuit. Data are presented as mean ± SD, n = 3 biologically independent samples. Statistical significance was calculated via two-tailed unpaired t test. ~~*P < 0.5; **P < 0.1; ***P < 0.01; ****P < 0.001~~. Source data are provided as a Source Data file.

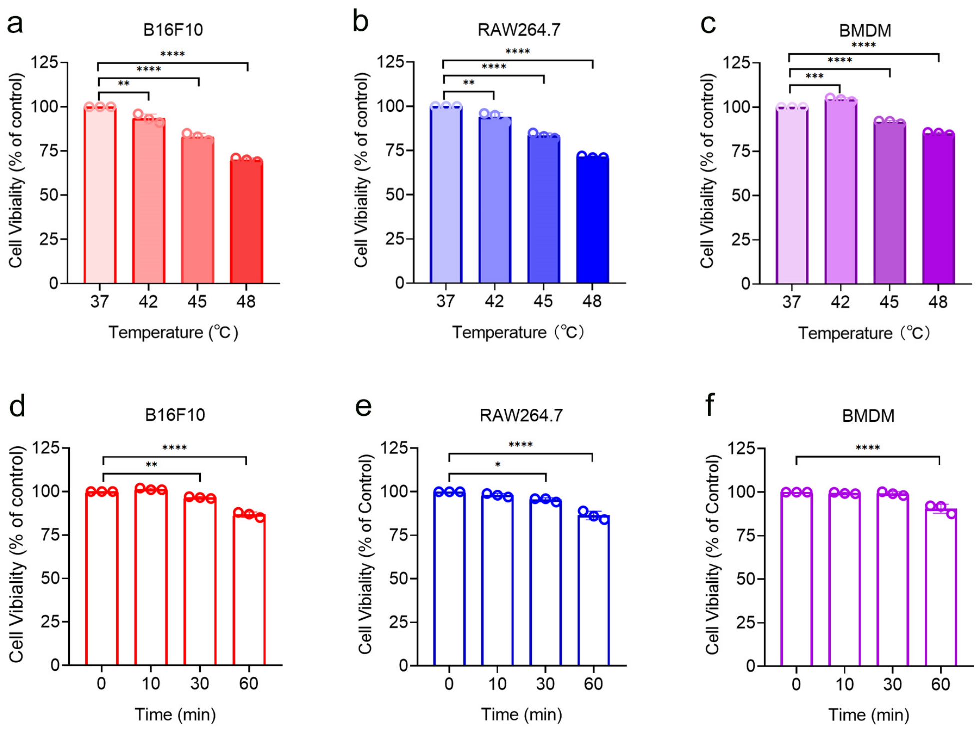

Supplementary Fig. 3 Cell viability of B16F10 or RAW264.7 or BMDM determined by CCK8 after wireless-controlled heating at different temperature for 30 min (a, b, c), or at 42 °C for different duration (d, e, f). Data are presented as mean ± SD, n = 3 biologically independent samples in a-f. Statistical significance was calculated via one-way analysis of variance (ANOVA) with a Dunnett's multiple comparisons test. ~~*P < 0.5; **P < 0. 1; ***P < 0.01; ****P < 0.001~~. Source data are provided as a Source Data file.

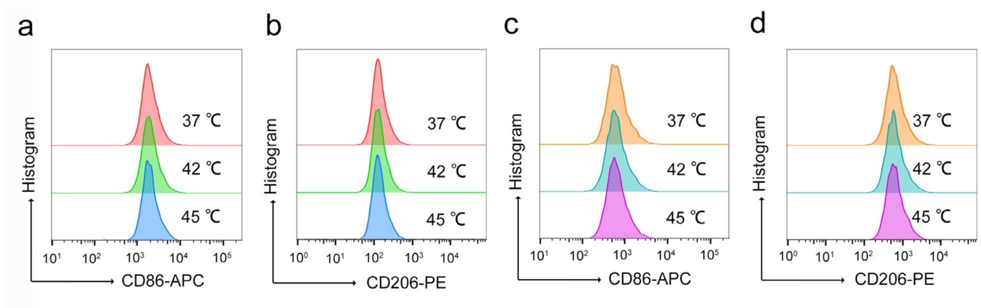

Supplementary Fig. 4 Flow cytometry analysis of the M1 polarization (a) and the M2 polarization (b) of BMDM after the heating at different temperature for 30 min. Flow cytometry analysis of the M1 polarization (c) and the M2 polarization (d) of IL4-treated BMDM after the heating at different temperature for 30 min. $CD86^{+}$ is the marker of M1 macrophages, while $CD206^{+}$ is the marker of M2 macrophages. This experiment was repeated three times independently with similar results.

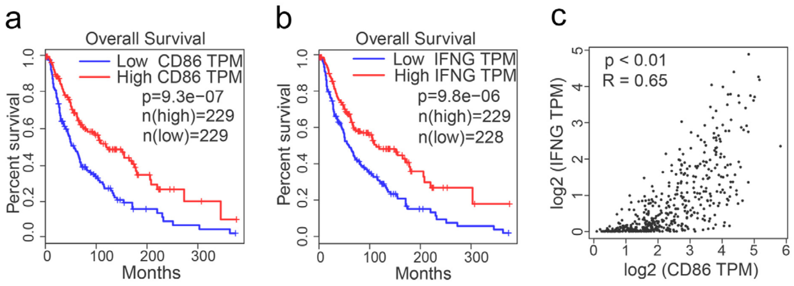

Supplementary Fig. 5 Overall survival of melanoma patients with different level of *CD86* (a) or *IFNG* (b), which was analyzed in GEPIA. (c) The correlation between *CD86* and *IFNG* in melanoma patients that were analyzed in GEPIA.

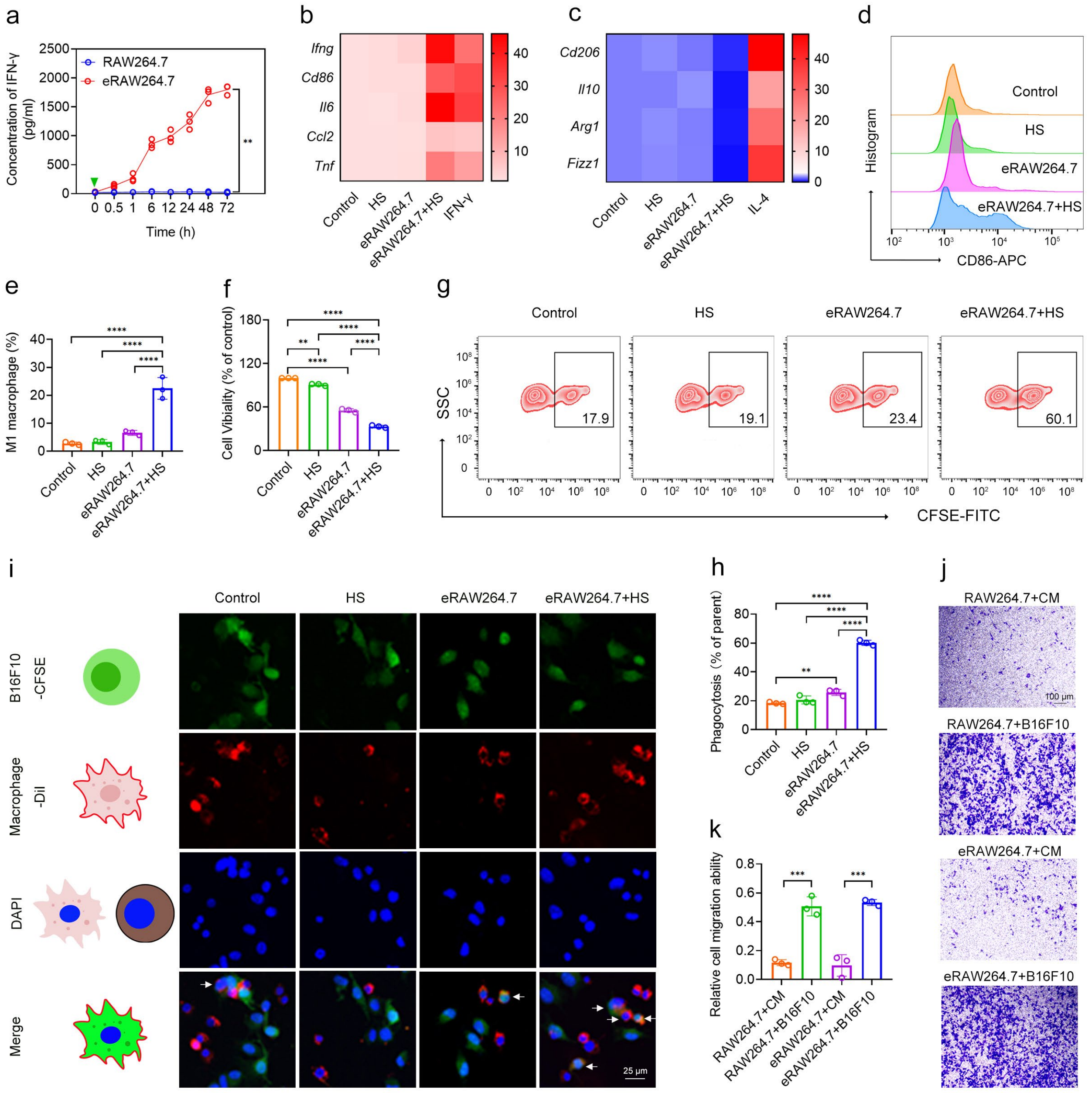

Supplementary Fig. 6 HS polarizes engineered RAW264.7 (eRAW264.7) into a M1 phenotype *in vitro*. (a) The secretion of IFN-γ by eRAW264.7 after HS at 42 °C for 30 min. RT-qPCR analysis of M1 macrophages markers (b) and M2 macrophages markers (c) after the indicated treatment. IFN-γ treated RAW264.7 and IL-4 treated RAW264.7 were used as positive controls. (d) Flow cytometry analysis and (e) quantitative analysis of $CD86^{+}$ macrophages in eRAW264.7 after the indicated treatment. (f) Pro-inflammatory cytokines-mediated inhibition of tumor proliferation was determined by CCK8 assay with the indicated treatment. (g) Flow cytometry and (h) quantitative analysis of the phagocytosis of RAW264.7 after the specified treatment. (i) Fluorescence images of the phagocytosis by RAW264.7. B16F10 cells were labeled with CFSE, the cell membrane of RAW264.7 was labeled with DiI, and the nuclei of both cells were stained with DAPI. The white arrows point to the B16F10 cell phagocytized by macrophages. (j) The tumor-targeting tropism was evaluated by the number of RAW264.7 that migrates through the semipermeable membrane. (k) Quantitative analysis of cell migration ability in (j) after the specified treatment. CM stands for culture medium. Data are presented as mean ± SD, n = 3 biologically independent samples in a-j. Statistical significance was calculated via two-tailed paired t test in a, one-way analysis of variance (ANOVA) with a Tukey's multiple comparisons test in e-h and two-tailed unpaired t test in a k. ~~*P < 0.5; **P < 0. 1; ***P < 0.01; ****P < 0.001~~. Source data are provided as a Source Data file.

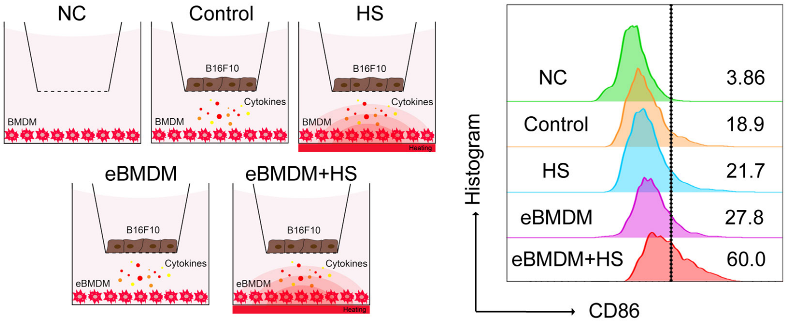

Supplementary Fig. 7 Flow cytometric analysis of $CD86^{+}$ macrophages in eBMDM after the indicated treatment. This experiment was repeated three times independently with similar results.

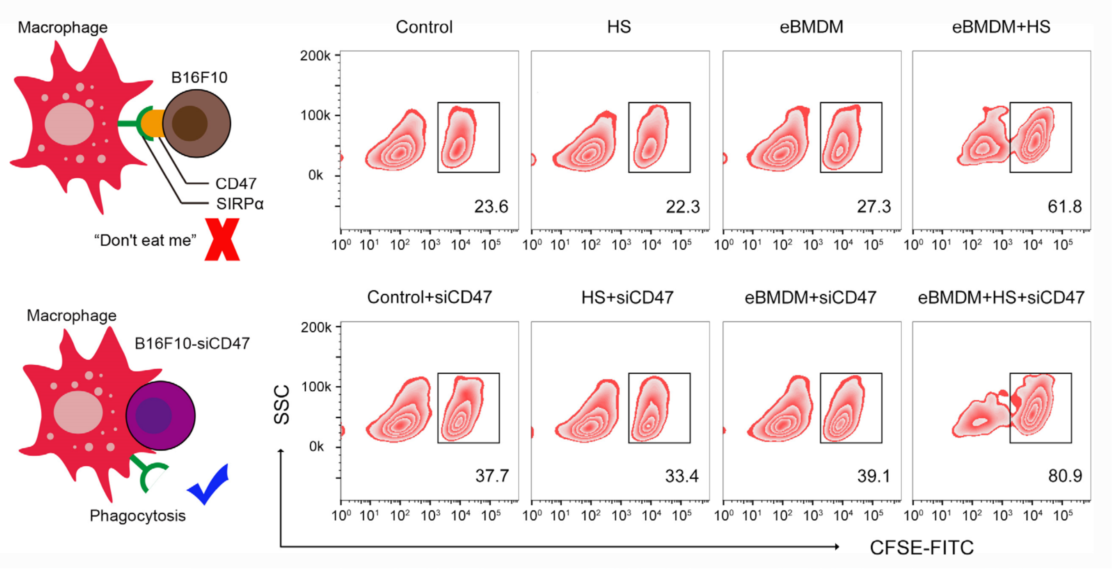

Supplementary Fig. 8 Flow cytometry analysis of the phagocytosis of BMDM in a co-culture system, in which DiL-labeled BMDM were cultured with B16F10 cells or CD47-knockdown B16F10 cells. This experiment was repeated three times independently with similar results.

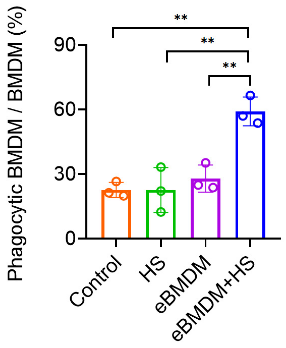

Supplementary Fig. 9 Quantitative analysis of the phagocytosis of BMDMs after the specified treatment in figure 2m. Data are presented as mean ± SD, n = 3 biologically independent samples. Statistical significance was calculated via one-way analysis of variance (ANOVA) with a Tukey's multiple comparisons test. ~~*P < 0.5; **P < 0. 1; ***P < 0.01; ****P < 0.001~~. Source data are provided as a Source Data file.

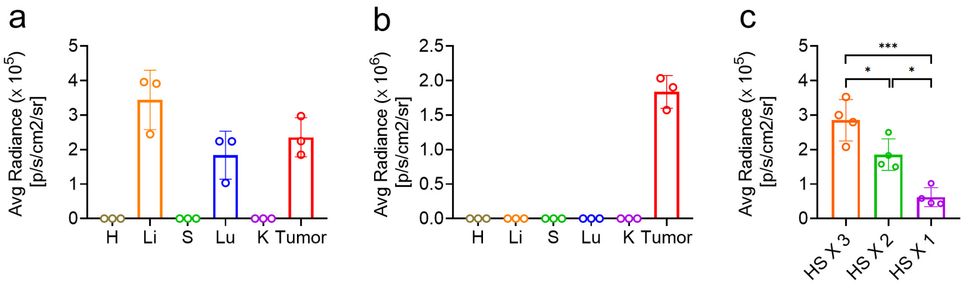

Supplementary Fig. 10 (a) Quantitative analysis of fluorescence in figure 3a. (b) Quantitative analysis of bioluminescence in figure 3b. (c) Quantitative analysis of bioluminescence in figure 3c. Data are presented as mean ± SD, n = 3 biologically independent samples in a and b, n = 4 biologically independent samples in c. Statistical significance was calculated via one-way analysis of variance (ANOVA) with a Tukey's multiple comparisons test in c. ~~$*P < 0.5$; $**P < 0. 1$; $***P < 0.01$; $****P < 0.001$~~. Source data are provided as a Source Data file.

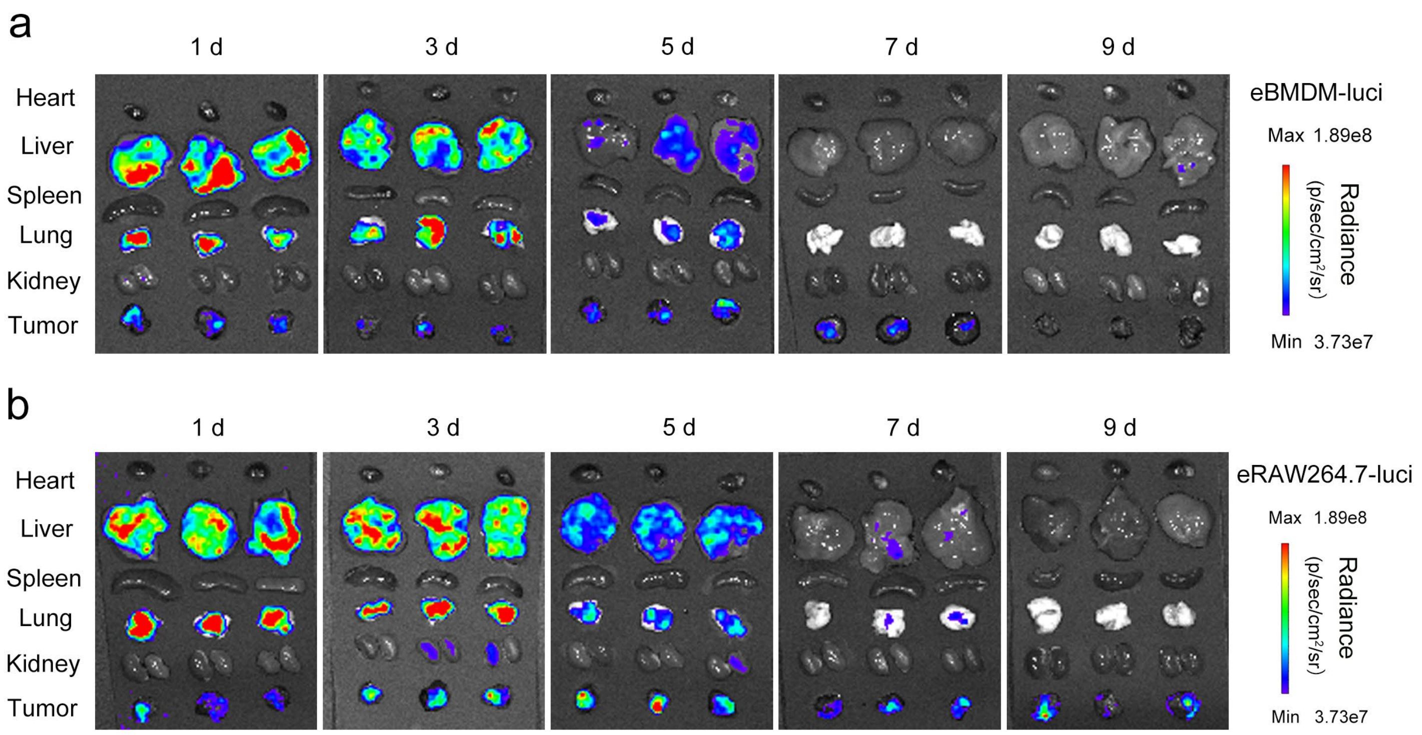

Supplementary Fig. 11 Survival time of eBMDM and eRAW264.7 *in vivo*. Biodistribution and survival time of the eBMDM (a) and eRAW264.7 (b) in major organs and the tumor tissue at different time points after *i.v* injection of luciferase-expressed eBMDM or eRAW264.7. n = 3 biologically independent samples.

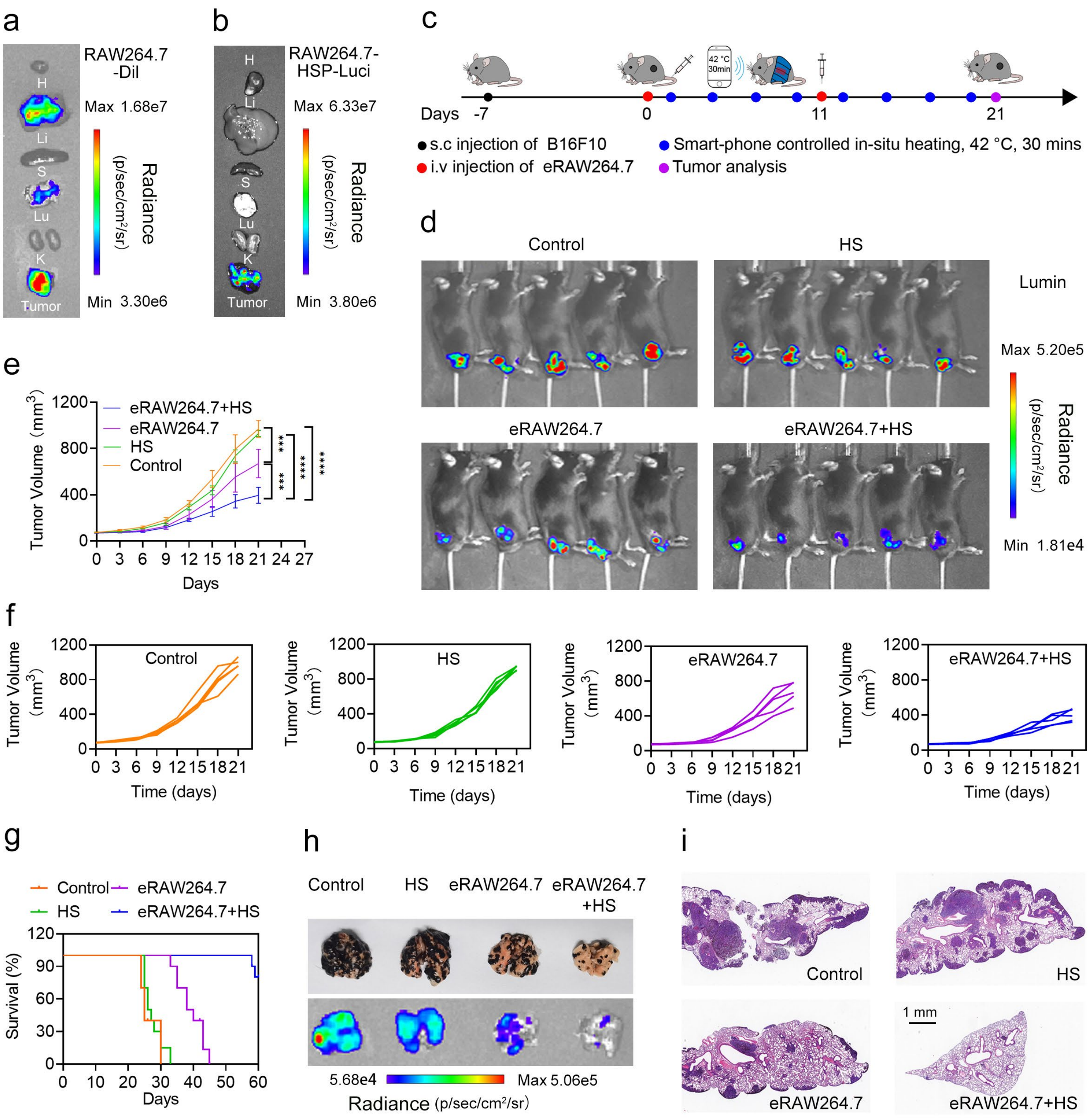

Supplementary Fig. 12 eRAW264.7-mediated treatment *in vivo* controlled by iWarm. (a) Fluorescence image of the eRAW264.7 distribution in major organs and tumor tissue after intravenous (*i.v*) injection of DiI-labelled eRAW264.7 for 24 h. (b) Bioluminescence image of major organs and tumor tissue after *i.v* injection of HSP-luciferase-expressing eRAW264.7 for 24 h, followed by locoregional hyperthermia in tumor tissue. (c) Illustration of B16F10 tumor therapy *in vivo* with eRAW264.7 via remote control of locoregional hyperthermia. (d) *In vivo* bioluminescence images of mice receiving the indicated treatment at day 21. The mice were inoculated with B16F10 tumor cells stably expressing luciferase. (e) Average and (f) individual tumor growth curves after the specified treatment. (g) Survival curves of mice after the specified treatment. (h) Bioluminescence image of lung metastatic nodules of the B16F10 tumors after the treatment. (i) H&E staining of lung metastatic nodules of B16F10 tumor after the treatment. Data are presented as mean ± SD, n = 3 biologically independent samples in a-b, n = 5 biologically independent samples in c-f, n = 10 biologically independent samples in g and n = 3 biologically independent samples in h-i. Statistical significance was calculated via one-way analysis of variance (ANOVA) with a Tukey's multiple comparisons test in e. ~~*P < 0.5; **P < 0. 1; ***P < 0.01; ****P < 0.001~~. Source data are provided as a Source Data file.

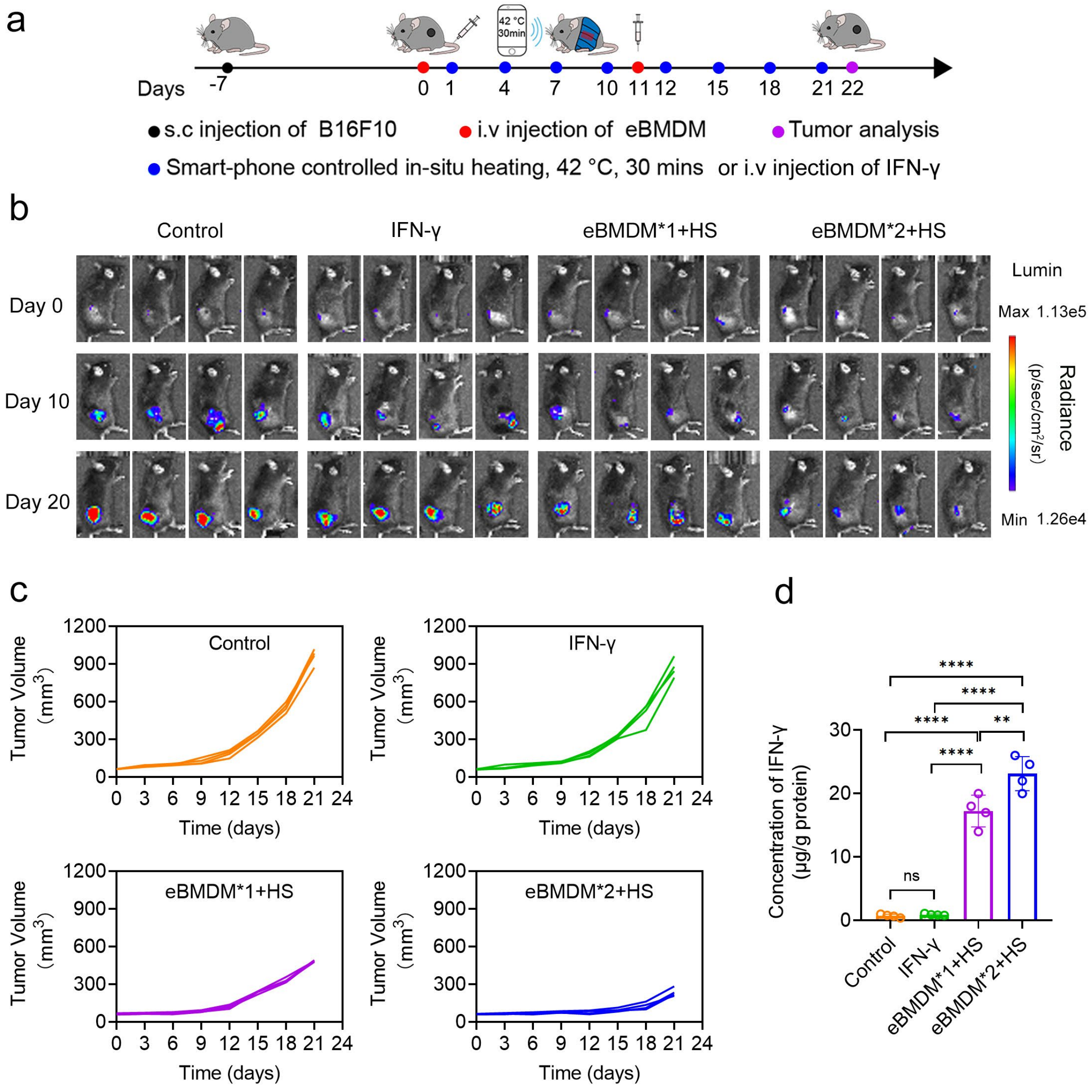

Supplementary Fig. 13 eBMDM-mediated treatment *in vivo* controlled by iWarm. (a) Illustration of B16F10 tumor therapy *in vivo* with eBMDM via the remote control of locoregional hyperthermia or IFN-γ. (b) *In vivo* bioluminescence images of mice receiving the indicated treatment at day 0, day 10 and day 20. The mice were inoculated with B16F10 tumor cells stably expressing luciferase. (c) Individual tumor growth curves after the indicated treatment. (d) IFN-γ level detected in the tumor tissue after the treatment. Data are presented as mean ± SD, n = 4 biologically independent mice or samples in a-d. Statistical significance was calculated via one-way analysis of variance (ANOVA) with a Tukey's multiple comparisons test in d. ~~*P < 0.5; **P < 0. 1; ***P < 0.01; ****P < 0.001~~. Source data are provided as a Source Data file.

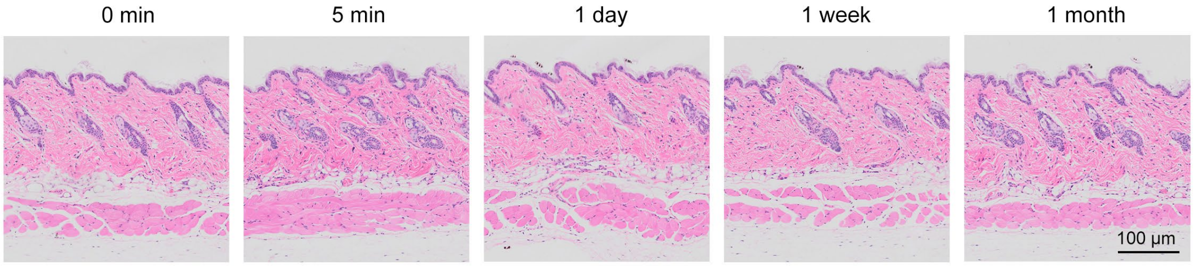

Supplementary Fig. 14 H&E staining of mouse skin slice at different time points after using iWarm-enabled locoregional hyperthermia at 42 ℃ for 30 min every day. This experiment was repeated three times independently with similar results.

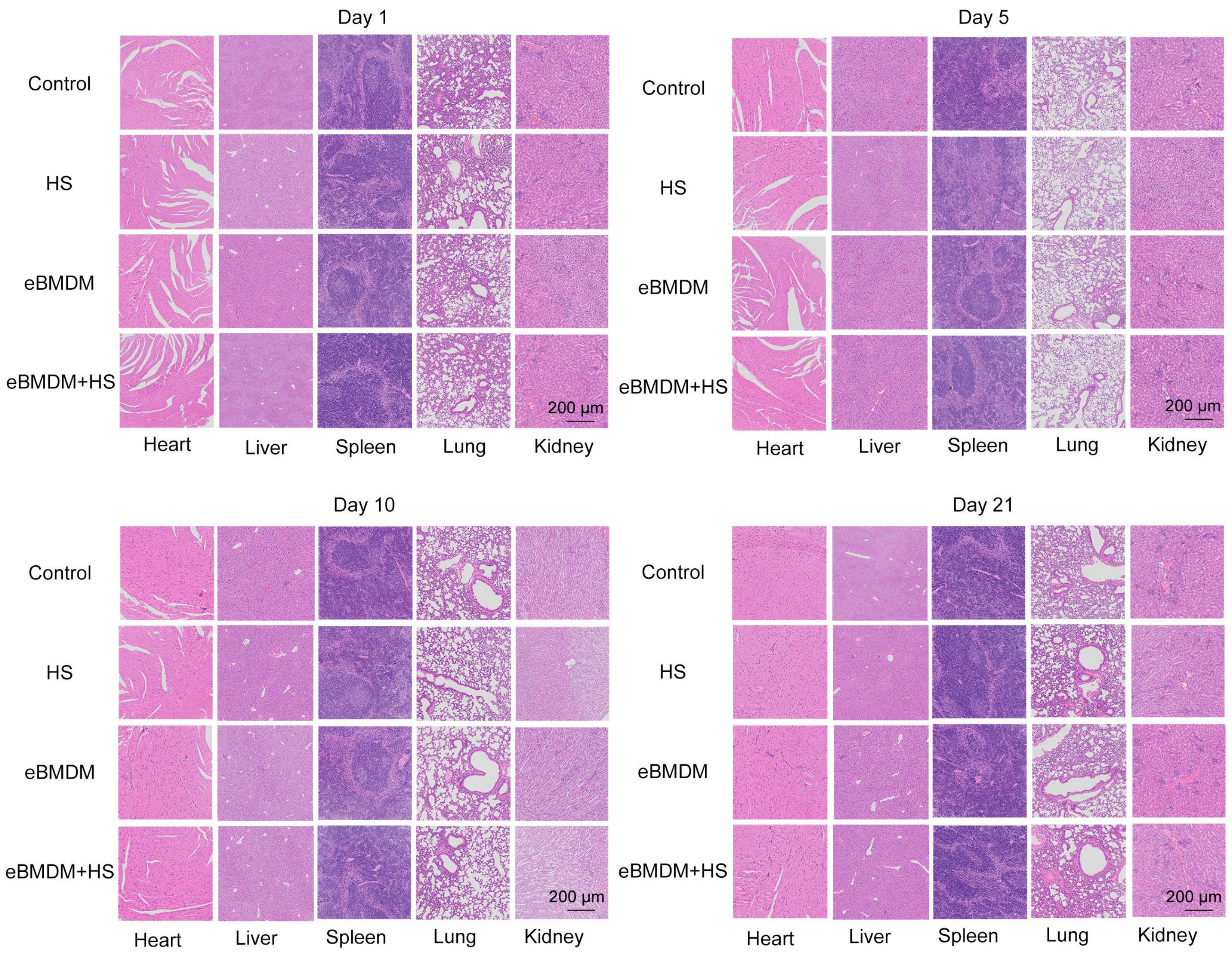

Supplementary Fig. 15 H&E staining of major organs at different time points after the indicated treatment. This experiment was repeated three times independently with similar results.

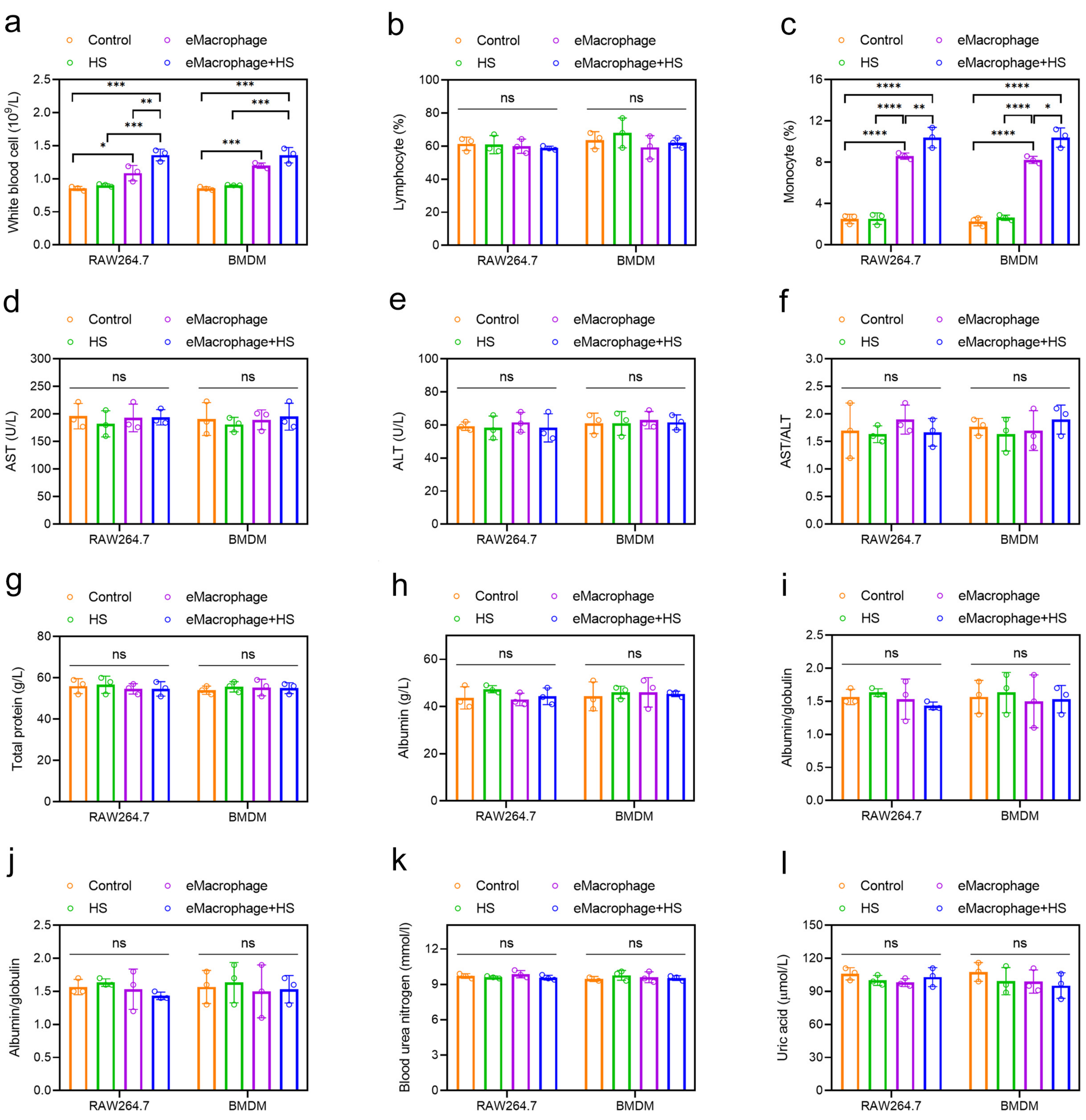

Supplementary Fig. 16 Hematological evaluation after the indicated treatment. The counts of (a) white blood cell, (b) lymphocyte, (c) monocyte, (d) AST (aspartate aminotransferase), (e) ALT (alanine aminotransferase), (f) AST/ALT, (g) total protein, (h) albumin, (i) globulin, (j) albumin/globulin, (k) blood urea nitrogen, (l) uric acid in serum at day 21. Data represent mean ± SD, n = 3 biologically independent samples in a-l. Statistical significance was calculated via one-way analysis of variance (ANOVA) with a Tukey's multiple comparisons test in a-l. ~~*P < 0.5; **P < 0. 1; ***P < 0.01; ****P < 0.001~~; ns for not significant. Source data are provided as a Source Data file.

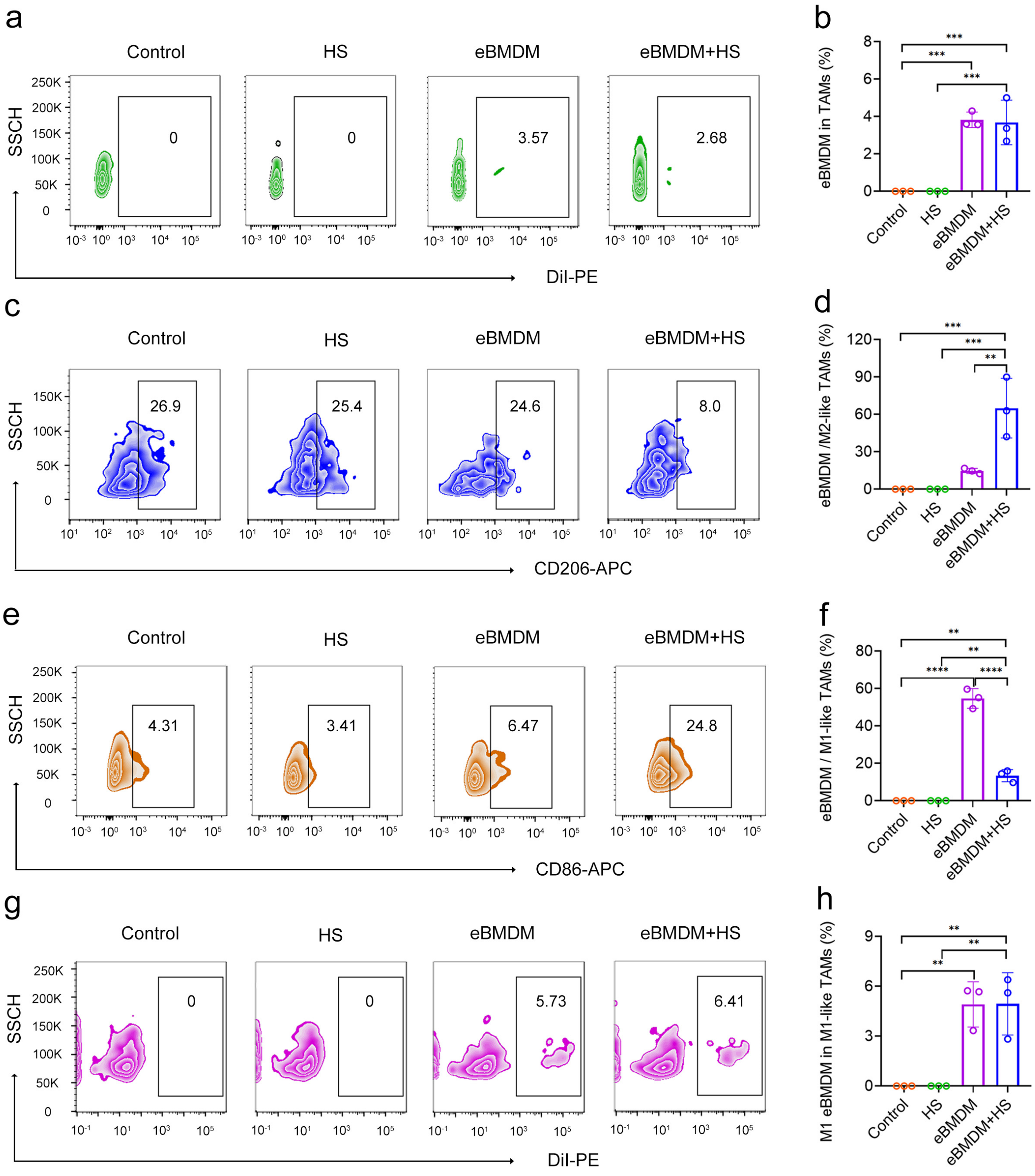

Supplementary Fig. 17 (a) Flow cytometry analysis the infiltration of eBMDM in melanoma tissue after the indicated treatment. DiL$^+$ is the marker of eBMDM. (b) Quantitative analysis the eBMDM ratio in TAMs in a. (c) Flow cytometry analysis M2-like TAMs in tumor tissues after the indicated treatment. CD206$^+$ is the marker of M2-like TAMs. (d) Quantitative analysis the percentage of adoptive eBMDM in TAMs. (e) Flow cytometry analysis the M1-like TAMs in tumor tissues after the indicated treatment. CD86$^+$ is the marker of M1-like TAMs. (f) Quantitative analysis the percentage of adoptive eBMDM in M1-like TAMs. (g) Flow cytometry analysis the ratio of M1 eBMDMs in M1-like TAMs after the indicated treatment. (h) Quantitative analysis the M1 eBMDM ratio in M1-like TAMs in g. Data are presented as mean ± SD, n = 3 biologically independent samples in a-h. Statistical significance was calculated via one-way analysis of variance (ANOVA) with a Tukey's multiple comparisons test in b, d, f, h. ~~*P < 0.5; **P < 0. 1; ***P < 0.01; ****P < 0.001~~. Source data are provided as a Source Data file.

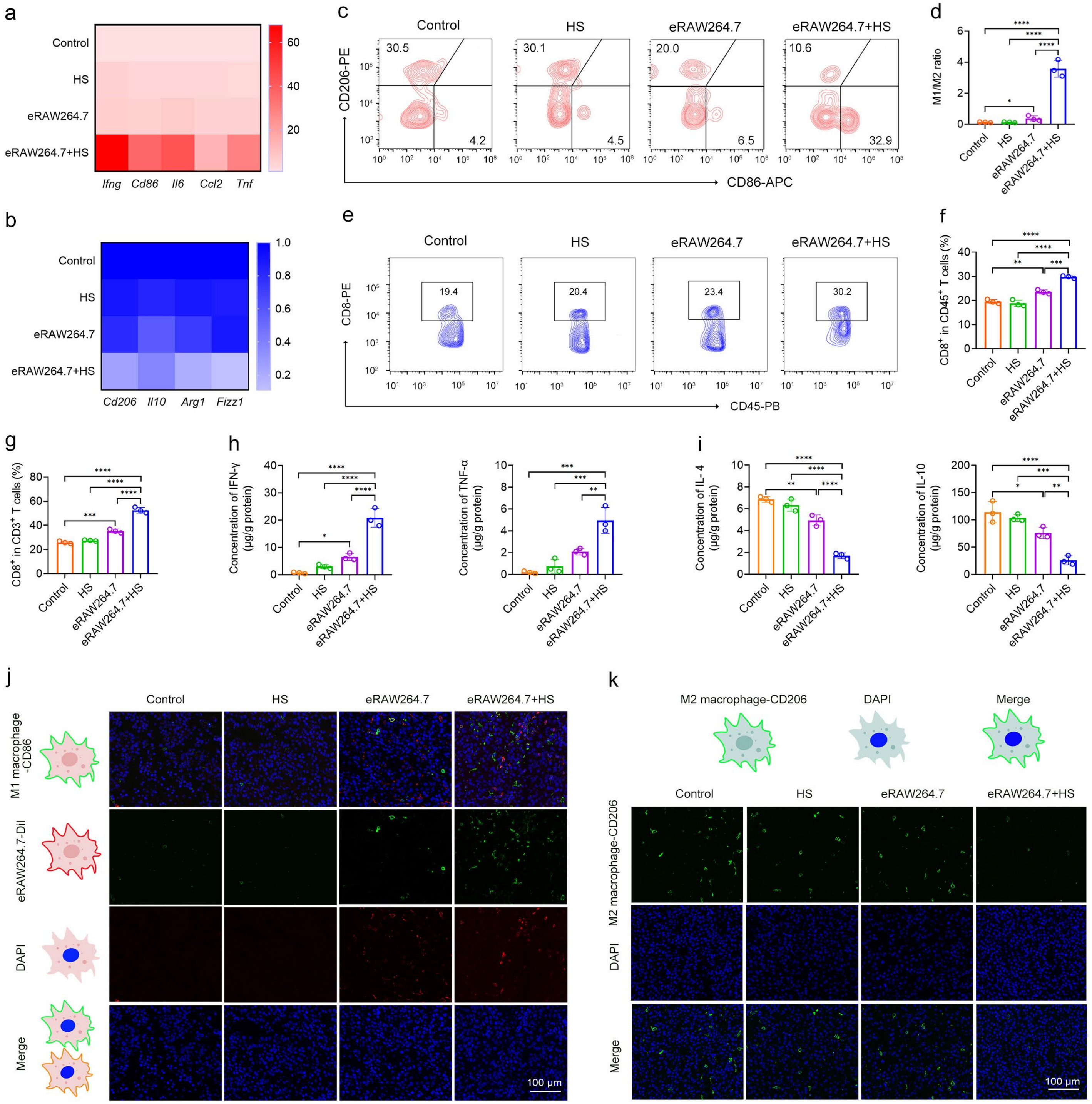

Supplementary Fig. 18 Wireless controlled iWarm mediated the polarization of eRAW264.7 and repolarization TAMs into a M1 phenotype, which triggered robust antitumor immunity *in vivo*. RT-qPCR analysis of M1 (a) and M2 (b) macrophages markers in tumor tissues after the indicated treatment. (c) Flow cytometry analysis of the polarization of macrophages in tumor tissues after the indicated treatment. $CD86^+$ is the marker of M1 macrophages, while $CD206^+$ is the marker of M2 macrophages. (d) Quantitative analysis of M1/M2 ratio of macrophages in (c). (e) Flow cytometry analysis of $CD8^+$ T cells in tumor tissues after eRAW264.7 treatment with or without heat shock. Quantitative analysis of the ratio of $CD8^+$ T cells in (f) $CD45^+$ cells and (g) $CD3^+$ cells in tumor tissues. (h) IFN-γ and TNF-α levels in tumor tissues collected from mice after the indicated treatment. (i) IL-4 and IL-10 levels in tumor tissues collected from mice after the indicated treatment. (j) Multiplex IHC images of M1 macrophage and eRAW264.7 infiltration in tumor tissues. In merged figures, DiI-positive and CD86-positive cells (orange) represent M1 eRAW264.7, CD86-positive only cells (green) represent re-polarized TAMs. (k) IHC images of M2 macrophage infiltration in tumor tissues. Data are presented as mean ± SD, n = 3 biologically independent samples in a-k. Statistical significance was calculated via one-way analysis of variance (ANOVA) with a Tukey's multiple comparisons test in d and f-i. ~~*$P < 0.5$; **$P < 0.1$; ***$P < 0.01$; ****$P < 0.001$~~. Source data are provided as a Source Data file.

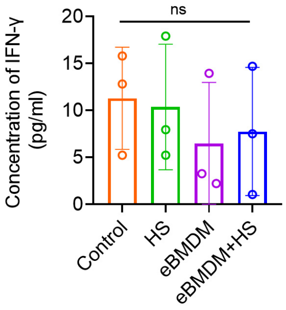

Supplementary Fig. 19 IFN-γ levels in serum collected from mice after the indicated treatment. Data represent mean ± SD, n = 3 biologically independent samples. Statistical significance was calculated via one-way analysis of variance (ANOVA) with a Tukey's multiple comparisons test, ns for not significant. Source data are provided as a Source Data file.

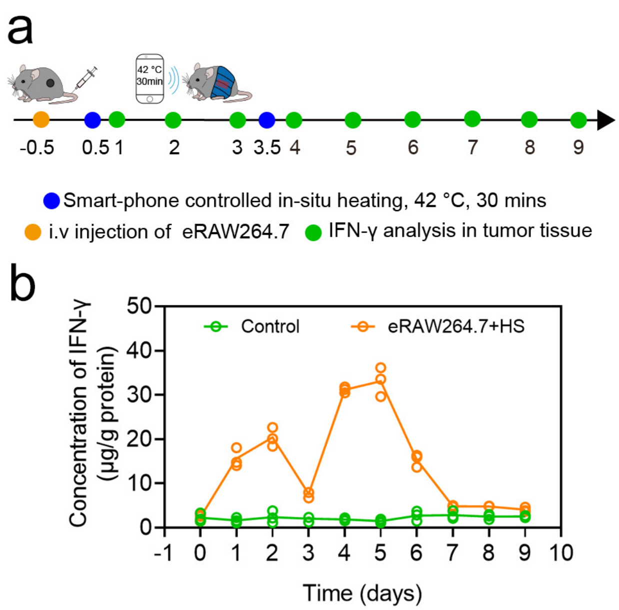

Supplementary Fig. 20 (a) Illustration of B16F10 tumor therapy *in vivo* with eRAW264.7 via remote control of locoregional hyperthermia. (b) IFN-γ level detected in tumor tissue *in vivo*. n = 3 biologically independent samples. Source data are provided as a Source Data file.

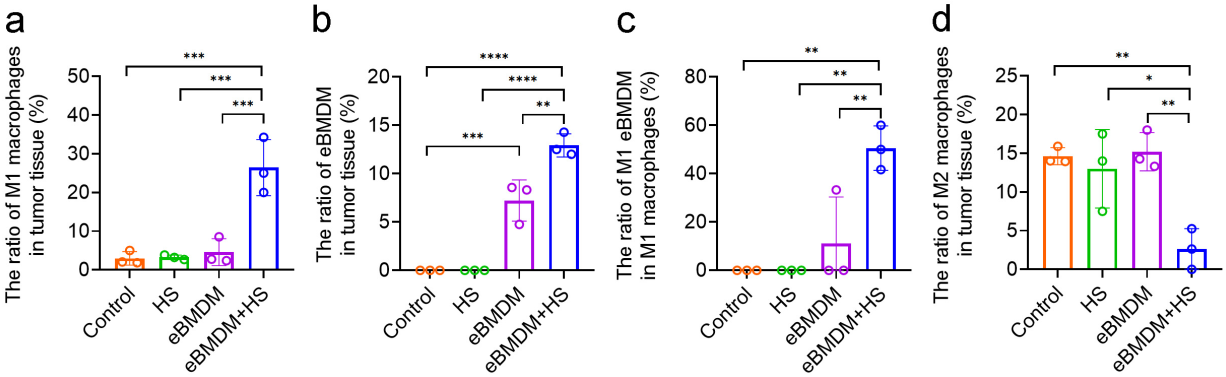

Supplementary Fig. 21 Quantitative analysis of the ratio of M1 macrophages in tumor tissue (a), eBMDM in tumor tissue (b) and the ratio of M1 eBMDM in M1 macrophages (c) after the specified treatment in Figure 4j. (d) Quantitative analysis of the ratio of M2 macrophages after the specified treatment in Figure 4k. Data are presented as mean ± SD, n = 3 biologically independent samples in a-d. Statistical significance was calculated via one-way analysis of variance (ANOVA) with a Tukey's multiple comparisons test in a-d. ~~*P < 0.5; **P < 0. 1; ***P < 0.01; ****P < 0.001~~. Source data are provided as a Source Data file.

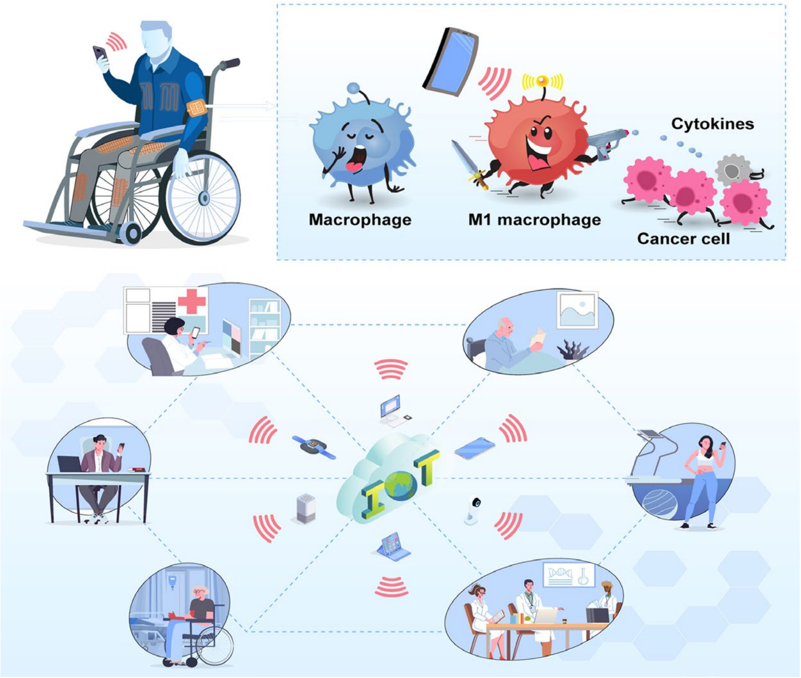

Supplementary Fig. 22 The potential application of wireless-controlled engineered macrophages in telemedicine.

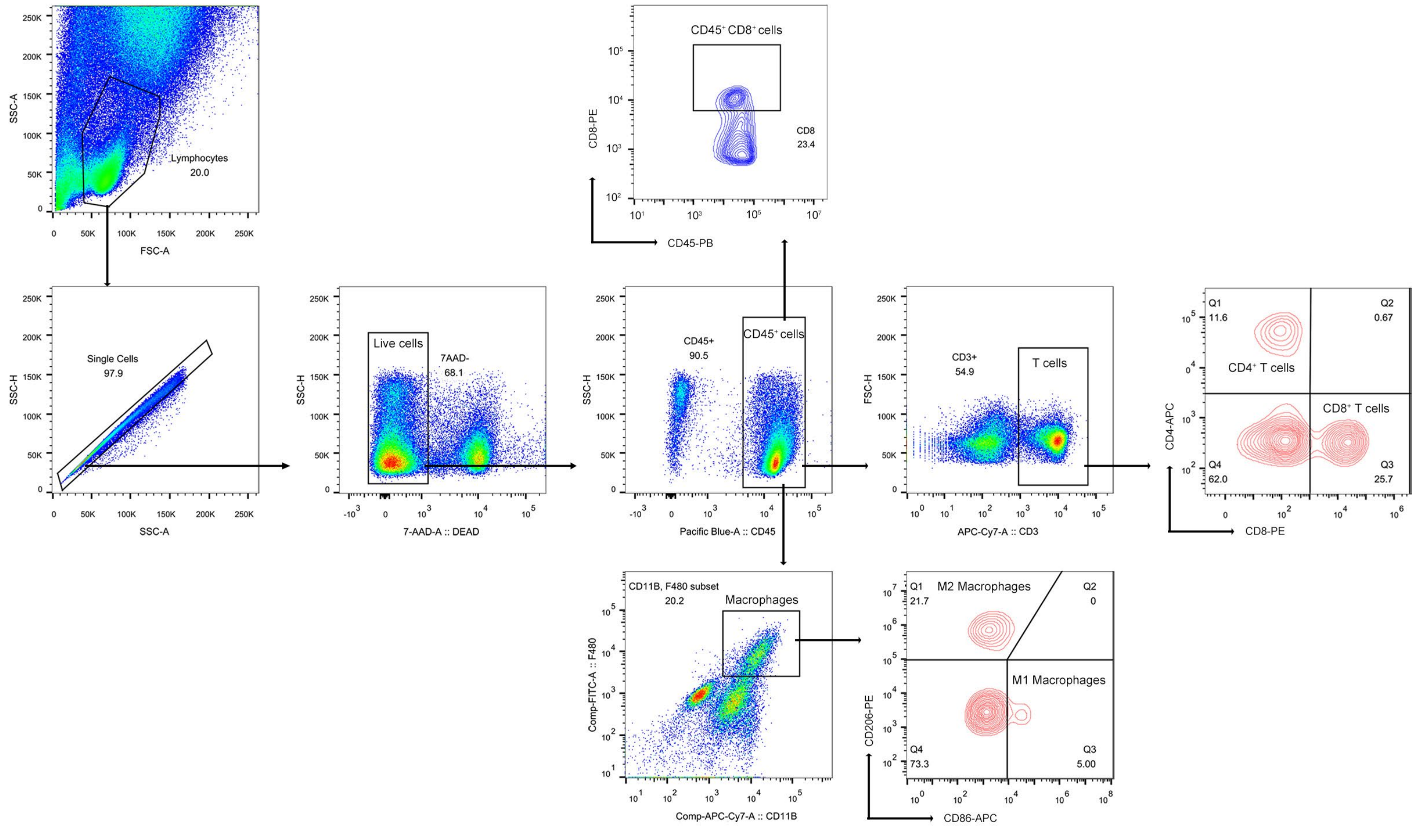

Supplementary Fig. 23 Gating strategy of CD8⁺ T cells within CD45⁺ cells or CD3⁺ T cells and gating strategy of M1 and M2 macrophages.

Supplementary Table

Supplementary Table 1. Primer sequences for PCR amplification and sgRNA synthesis.

Primer names	Sequences (5'-3')
Ifng-F	ATGAACGCTACACACTGCATC
Ifng-R	CCATCCTTTTGCCAGTTCCTC
Il6-F	TCTATACCACTTCACAAGTCGGA
Il-6-R	GAATTGCCATTGCACAACTCTTT
Tnf-F	CAGGCGGTGCCTATGTCTC
Tnf-R	CGATCACCCCGAAGTTCAGTAG
Cd86-F	GAGCTGGTAGTATTTTGGCAGG
Cd86-R	GGCCCAGGTACTTGGCATT
Ccl2-F	TTAAAAACCTGGATCGGAACCAA
Ccl2-R	GCATTAGCTTCAGATTTACGGGT
Arg1-F	CTCCAAGCCAAAGTCCTTAGAG
Arg1-R	AGGAGCTGTCATTAGGGACATC
Cd206-F	CTCTGTTCAGCTATTGGACGC
Cd206-R	CGGAATTTCTGGGATTCAGCTTC
Il10-F	CTTACTGACTGGCATGAGGATCA
Il10-R	GCAGCTCTAGGAGCATGTGG
Fizz1-F	CCAATCCAGCTAACTATCCCTCC
Fizz1-R	ACCCAGTAGCAGTCATCCCA
Actb-F	GGCTGTATTCCCCTCCATCG
Actb-R	CCAGTTGGTAACAATGCCATGT
Ifng-sgRNA-1-F	CACCGTTACTTTGCATTACAGCTA
Ifng-sgRNA-1-R	AAACTAGCTGTAATGCAAAGTAAC
Ifng-sgRNA-2-F	CACCGTTGGGGACGGCTGAATAAA
Ifng-sgRNA-2-R	AAACTTTATTCAGCCGTCCCCAAC
Ifng-sgRNA-3-F	CACCGAATCCCACAAGAATGGCAC
Ifng-sgRNA-3-R	AAACGTGCCATTCTTGTGGGATTC
