# Supplementary material for: Proinflammatory polarization of engineered heat-inducible macrophages reprogram the tumor immune microenvironment during cancer immunotherapy
Source: Nat Commun. 2024 Mar 15;15:2270. doi: 10.1038/s41467-024-46210-1 (PMC10943244; doi:10.1038/s41467-024-46210-1)
Supplement: Supplementary file 3 — Description of Additional Supplementary Files [file 41467_2024_46210_MOESM3_ESM.pdf]

Description of Additional Supplementary Files

Supplementary Movie 1: The movie for remote control of iWarm. Once turning on iWarm from the Web APP (https://www.we-share.work/heating/index), it quickly heated up to the preset temperature and cooled down to room temperature at the end of preset time. The repeated heating and cooling of five cycles resulted in a similar temperature fluctuation, which can be also simultaneously monitored through the Web APP.

Supplementary Movie 2: The movie for smart phone-controlled iWarm. Once turning on iWarm from the APP, it quickly heated up to the preset temperature and cooled down to room temperature at the end of preset time, which can be also simultaneously monitored through the APP.
