# Supplementary material for: Proinflammatory polarization of engineered heat-inducible macrophages reprogram the tumor immune microenvironment during cancer immunotherapy
Source: Nat Commun. 2024 Mar 15;15:2270. doi: 10.1038/s41467-024-46210-1 (PMC10943244; doi:10.1038/s41467-024-46210-1)
Supplement: Supplementary file 6 — Reporting Summary [file 41467_2024_46210_MOESM6_ESM.pdf]

nature portfolio

Corresponding author(s): Yuan Ping

Last updated by author(s): Jan 22, 2024

Reporting Summary

Nature Portfolio wishes to improve the reproducibility of the work that we publish. This form provides structure for consistency and transparency in reporting. For further information on Nature Portfolio policies, see our Editorial Policies and the Editorial Policy Checklist.

Statistics

For all statistical analyses, confirm that the following items are present in the figure legend, table legend, main text, or Methods section.

n/a	Confirmed	
☐	☒	The exact sample size (*n*) for each experimental group/condition, given as a discrete number and unit of measurement
☐	☒	A statement on whether measurements were taken from distinct samples or whether the same sample was measured repeatedly
☐	☒	The statistical test(s) used AND whether they are one- or two-sided<br>*Only common tests should be described solely by name; describe more complex techniques in the Methods section.*
☒	☐	A description of all covariates tested
☒	☐	A description of any assumptions or corrections, such as tests of normality and adjustment for multiple comparisons
☐	☒	A full description of the statistical parameters including central tendency (e.g. means) or other basic estimates (e.g. regression coefficient) AND variation (e.g. standard deviation) or associated estimates of uncertainty (e.g. confidence intervals)
☐	☒	For null hypothesis testing, the test statistic (e.g. *F*, *t*, *r*) with confidence intervals, effect sizes, degrees of freedom and *P* value noted<br>*Give P values as exact values whenever suitable.*
☒	☐	For Bayesian analysis, information on the choice of priors and Markov chain Monte Carlo settings
☒	☐	For hierarchical and complex designs, identification of the appropriate level for tests and full reporting of outcomes
☒	☐	Estimates of effect sizes (e.g. Cohen's *d*, Pearson's *r*), indicating how they were calculated

Our web collection on statistics for biologists contains articles on many of the points above.

Software and code

Policy information about availability of computer code

Data collection	Flow cytometry: BD LSRFortessa; Confocal microscopy: Olympus FV3000; PCR: Thermo ABI QuantStudio 6; H&E staining images: Olympus Virtual digital slice scanning system VS120; Small animal live fluorescence image analysis system: Caliper IVIS Spectrum.
Data analysis	All data and figures in this paper were analyzed and plotted by Graphpad prism 8.0, FlowJo V10, ImageJ 1.53, Photoshop 2023, Illustrator 2023 and GEPIA2 browser.

For manuscripts utilizing custom algorithms or software that are central to the research but not yet described in published literature, software must be made available to editors and reviewers. We strongly encourage code deposition in a community repository (e.g. GitHub). See the Nature Portfolio guidelines for submitting code & software for further information.

Data

Policy information about availability of data

All manuscripts must include a data availability statement. This statement should provide the following information, where applicable:

- Accession codes, unique identifiers, or web links for publicly available datasets
- A description of any restrictions on data availability
- For clinical datasets or third party data, please ensure that the statement adheres to our policy

All data generated or analyzed during this study are included in this article and its supplemental materials. The correlation of gene expression with prognostic outcome were analyzed by GEPIA2 browser (http://gepia2.cancer-pku.cn). Source data are provided with this paper.

Research involving human participants, their data, or biological material

Policy information about studies with human participants or human data. See also policy information about sex, gender (identity/presentation), and sexual orientation and race, ethnicity and racism.

Reporting on sex and gender	No
Reporting on race, ethnicity, or other socially relevant groupings	No
Population characteristics	n/a
Recruitment	n/a
Ethics oversight	n/a

Note that full information on the approval of the study protocol must also be provided in the manuscript.

Field-specific reporting

Please select the one below that is the best fit for your research. If you are not sure, read the appropriate sections before making your selection.

☒ Life sciences ☐ Behavioural & social sciences ☐ Ecological, evolutionary & environmental sciences

For a reference copy of the document with all sections, see nature.com/documents/nr-reporting-summary-flat.pdf

Life sciences study design

All studies must disclose on these points even when the disclosure is negative.

Sample size	No sample size calculation was performed. Instead, sample sizes were chosen based on previously published works of a similar nature (Proc Natl Acad Sci U S A. 2020;117(5):2395-2405; Sci Adv. 2020;6(18):eaaz6579; Nat Commun. 2022;13(1):3419; Nat Nanotechnol. 2023;18(8):933-944). Sample sizes are clearly reported in the Figure Legend. In addition, we adhered to sample size requirements necessary for determining statistical significance.
Data exclusions	No data was excluded.
Replication	All experiments were replicated independently for at least 3 times, and the detailed information was provided in corresponding figure legends.
Randomization	Animal groups were randomized by body weight. In the other experiments, samples were randomly assigned to experimental groups.
Blinding	For all experiments, processing was performed without blinding to ensure correct treatments are made, but analysis was performed with blinding.

Reporting for specific materials, systems and methods

We require information from authors about some types of materials, experimental systems and methods used in many studies. Here, indicate whether each material, system or method listed is relevant to your study. If you are not sure if a list item applies to your research, read the appropriate section before selecting a response.

Materials & experimental systems

n/a	Involved in the study	
☐	☒	Antibodies
☐	☒	Eukaryotic cell lines
☒	☐	Palaeontology and archaeology
☐	☒	Animals and other organisms
☒	☐	Clinical data
☒	☐	Dual use research of concern
☒	☐	Plants

Methods

n/a	Involved in the study	
☒	☐	ChIP-seq
☐	☒	Flow cytometry
☒	☐	MRI-based neuroimaging

Antibodies

Antibodies used	The following primary antibodies were used for flow cytometry:<br>7-AAD Viability Staining Solution (420403, Biolegend);<br>Brilliant Violet 421 anti-mouse CD45 Antibody (103134, clone number: 30-F11, Biolegend);<br>Anti-mouse CD3-APC/Cy7 (100221, clone number: 17A2, Biolegend);<br>Anti-mouse CD4-APC (100412, clone number: GK1.5, Biolegend);<br>Anti-mouse CD8-PE (100708, clone number: 53-6.7, Biolegend);<br>Anti-mouse CD11b-APC/Cy7 (101226, clone number: M1/70, Biolegend);<br>Anti-mouse F4/80-FITC (123107, clone number: BM8, Biolegend);<br>Anti-mouse CD86-APC (105011, clone number: GL-1, Biolegend);<br>Anti-mouse CD206-PE (141706, clone number: C068C2, Biolegend);<br>Anti-mouse CD206-APC (142707, clone number: C068C2, Biolegend).<br><br>The following primary antibodies were used for immunofluorescent staining:<br>Anti-mouse CD86 Rabbit mAb (19589, clone number: E5W6H, Cell Signaling Technology);<br>Anti-mouse F4/80 Rabbit mAb (70076, clone number: D2S9R, Cell Signaling Technology);<br>Anti-mouse CD206 Rabbit mAb (24595, clone number: E6T5J, Cell Signaling Technology).
Validation	All antibodies were verified by the supplier and each lot has been quality tested. All the antibodies used are from commercial sources and have been validated by the vendors. Validation data are available on the manufacturer's website.<br>7-AAD Viability Staining Solution (https://www.biolegend.com/en-us/products/7-aad-viability-staining-solution-1649);<br>Brilliant Violet 421 anti-mouse CD45 Antibody (https://www.biolegend.com/en-us/products/brilliant-violet-421-anti-mouse-cd45-antibody-7253);<br>APC/Cyanine7 anti-mouse CD3 Antibody (https://www.biolegend.com/en-us/products/apc-cyanine7-anti-mouse-cd3-antibody-6068);<br>APC anti-mouse CD4 Antibody (https://www.biolegend.com/en-us/products/apc-anti-mouse-cd4-antibody-245);<br>PE anti-mouse CD8a Antibody (https://www.biolegend.com/en-us/products/pe-anti-mouse-cd8a-antibody-155);<br>APC/Cyanine7 anti-mouse/human CD11b Antibody (https://www.biolegend.com/en-us/products/apc-cyanine7-anti-mouse-human-cd11b-antibody-3930)<br>FITC anti-mouse F4/80 Antibody (https://www.biolegend.com/en-us/products/fitc-anti-mouse-f4-80-antibody-4067);<br>APC anti-mouse CD86 Antibody (https://www.biolegend.com/en-us/products/apc-anti-mouse-cd86-antibody-2896);<br>PE anti-mouse CD206 (MMR) Antibody (https://www.biolegend.com/en-us/products/pe-anti-mouse-cd206-mmr-antibody-7424);<br>APC anti-mouse CD206 (MMR) Antibody (https://www.biolegend.com/en-us/products/apc-anti-mouse-cd206-mmr-antibody-7425);<br>Anti-mouse CD86 Rabbit mAb (https://www.cellsignal.cn/products/primary-antibodies/cd86-e5w6h-rabbit-mab/19589?site-search-type=Products&N=4294956287&Ntt=19589&fromPage=plp&_requestid=273265);<br>Anti-mouse F4/80 Rabbit mAb (https://www.cellsignal.cn/products/primary-antibodies/f4-80-d2s9r-xp-rabbit-mab/70076?site-search-type=Products&N=4294956287&Ntt=70076&fromPage=plp&_requestid=275081);<br>Anti-mouse CD206 Rabbit mAb (https://www.cellsignal.cn/products/primary-antibodies/cd206-mrc1-e6t5j-xp-rabbit-mab/24595?site-search-type=Products&N=4294956287&Ntt=24595&fromPage=plp&_requestid=275345).

Eukaryotic cell lines

Policy information about cell lines and Sex and Gender in Research

Cell line source(s)	RAW 264.7 cells and B16F10 cells were purchased from the National Infrastructure of Biomedical Cell Line Resource (Beijing, China). Bone marrow derived macrophages (BMDMs) were generated from the bone marrow of C57BL/6 mice.
Authentication	The cell lines were authenticated using STR method by the suppliers.
Mycoplasma contamination	Mycoplasma testing was negative.
Commonly misidentified lines<br>(See ICLAC register)	No commonly misidentified cell lines were used in the study.

Animals and other research organisms

Policy information about studies involving animals; ARRIVE guidelines recommended for reporting animal research, and Sex and Gender in Research

Laboratory animals	8 weeks old C57BL/6 wild type mice were used for this study.
Wild animals	The study did not involve wild animals.
Reporting on sex	Mice of both sexes were used in this study.
Field-collected samples	The study did not involve samples collected from the field.
Ethics oversight	All animal treatments or procedures were carried out in accordance with the guidelines of the Laboratory Animal Welfare and Ethics Committee of Zhejiang University and approved by the Animal Ethics Committee of Sir Run Run Shaw Hospital, School of Medicine, Zhejiang University.

Note that full information on the approval of the study protocol must also be provided in the manuscript.

Plants

Seed stocks	n/a
Novel plant genotypes	n/a
Authentication	n/a

Flow Cytometry

Plots

Confirm that:

☒ The axis labels state the marker and fluorochrome used (e.g. CD4-FITC).

☒ The axis scales are clearly visible. Include numbers along axes only for bottom left plot of group (a 'group' is an analysis of identical markers).

☒ All plots are contour plots with outliers or pseudocolor plots.

☒ A numerical value for number of cells or percentage (with statistics) is provided.

Methodology

Sample preparation	The tumor tissues were enriched from mice, cut into small pieces, and digested in cell culture media supplementing with DNases (0.2 mg/mL, Sigma-Aldrich), collagenase D (1 mg/mL), and hyaluronidase (0.1 mg/mL) at 37 ℃ for 30 minutes, and were then filtered with 70-µm cell strainers. Then cells were stained with fluorescence-labeled antibodies.
Instrument	BD LSRFortessa was used for data collected.
Software	FlowJo V10 was used for data analysis.
Cell population abundance	Purity of isolated samples was determined by antibody stain and flow cytometry. Sample purity was greater than 90%.
Gating strategy	Gating was first based on FSC/SSC and singlet cells were gated for further analysis. The cell populations were then analyzed based on expression of markers. Gating was then based on positive level. T cells were gated as CD45+CD8+, CD45+CD3+CD4+ and CD45+CD3+CD8+, macrophages were gated as CD45+F4/80+CD11b+CD86+ (M1) and CD45+F4/80+CD11b+CD206+ (M2).

☒ Tick this box to confirm that a figure exemplifying the gating strategy is provided in the Supplementary Information.
